# Assessment Fit for (More Than One) Purpose? Reconciling the Dual Purposes of Assessments in Competency-Based Health Professions Education

**DOI:** 10.5334/pme.2592

**Published:** 2026-09-29

**Authors:** Michael S. Ryan, Aleda M. H. Chen, Heather Nichol, Sören Huwendiek, Denyse Richardson, Benjamin Kinnear, Warren J. Cheung, Claire Touchie, Fremen Chihchen Chou, David A. Turner, Anna Oswald, As members of the International Competency-Based Health Professions Education Collaborative

**Affiliations:** 1 Department of Pediatrics, University of Virginia School of Medicine, Charlottesville, Virginia, United States of America; 2 University of Arkansas Medical Sciences, Little Rock, Arkansas, United States of America; 3 Department of Medicine, Dalhousie University, Stan Cassidy Centre for Rehabilitation, Fredericton, New Brunswick, Canada; 4 Department for Assessment and Evaluation, Institute for Medical Education, University of Bern, Switzerland; 5 Department of Medicine, University of Alberta, Edmonton, Alberta, Canada; 6 Department of Pediatrics, University of Cincinnati College of Medicine, Cincinnati, Ohio, United States of America; 7 Department of Emergency Medicine, University of Ottawa, Ottawa, Ontario, Canada; 8 Centre for Innovation in Medical Education, University of Ottawa, Ottawa, Ontario, Canada; 9 School of Medicine, China Medical University, Taichung, Taiwan; 10 China Medical University Hospital, Taichung, Taiwan; 11 Innovation and Competency-Based Education, American Board of Pediatrics, Chapel Hill, NC, United States of America; 12 Division of Rheumatology, Department of Medicine, University of Alberta, Edmonton, Alberta, Canada

## Abstract

Assessments serve several purposes in competency-based medical education (CBME) and competency-based health professions education (CBHPE) programs. These purposes are commonly termed ‘assessment *for* learning’ and ‘assessment *of* learning.’ Historically, educational scholars have advised against using an assessment for multiple purposes, arguing that doing so undermines its primary purpose. However, at times, such an approach may be required from a pragmatic standpoint. In this Eye Opener, the authors consider the competing purposes of assessment in CBHPE programs from a practical lens. They propose specific strategies to maximize benefits and minimize risks of dual-purposing when such an approach is necessary. First, the authors describe the importance of setting the stage for dual-purposing by creating a culture of trust, which requires clear institutional commitment to learner orientation and faculty development, infrastructure, and appropriate channels of communication with all constituents. Second, data considerations are discussed. The authors focus on adoption of programmatic assessment principles, opportunities for frequent formative observations, and strategies for leveraging available technology. Finally, the focus turns to the learner, highlighting their essential function as a key agent in driving their own growth and development. Balancing learner- and supervisor-initiated assessments, implementing longitudinal coaching programs, and considering “ultra-low-stakes” assessments are identified as mechanisms to support learner agency and development. Taken together, these specific design conditions may be used to promote learner growth and acquisition of competence when practical constraints require assessment data to serve a dual purpose.

## Introduction

Assessment serves multiple purposes in health professions education [1]. These purposes may include determining a learner’s level of competence for a particular activity or domain, referred to as “assessment *of* learning” (sometimes referred to as *summative* assessment) or providing feedback to a learner to guide their future development, referred to as “assessment *for* learning” (sometimes referred to as *formative assessment*) [2,3]. Some have also expressed a third purpose, “assessment as learning” [4,5,6]. However, for the purpose of our discussion, we will focus on assessment for/of learning since these represent the predominant tensions in health professions assessment practice.

In this manuscript, we consider the prospect of using an assessment for more than one purpose, a practice commonly referred to as “dual purposing” [7,8,9]. Though this approach presents potential challenges [9], we argue that dual purposing can succeed in programs that intentionally address specific design conditions regarding culture, data structures, and learner agency. This manuscript thus serves as a practical guide, offering suggestions for educational leaders to optimize dual-purposing within their health professions education programs.

### Differentiating purpose from stakes

The purpose of assessment is often related to, but not synonymous with, the *stakes* of the assessment(s) [10]. Stakes refer to the implications of assessment(s) on a learner’s advancement within a program [11]. A classic high-stakes assessment is a certification examination, an assessment for which a passing score is often required for advancement. In contrast, low-stakes assessments are those that are of little consequence to the learner’s advancement. An example may include an ungraded reflective writing assignment that carries no weight in advancement decisions. A summary of key definitions commonly used in assessment programs is provided in Table 1.

**Table 1:** Definitions commonly used in describing assessment programs in health professions education.


TERM	DEFINITION	ILLUSTRATIVE EXAMPLE

**Purpose**		

Assessment for learning [2,3]	An assessment designed to serve primarily as a tool for learning.	Students are provided with an optional “self-assessment” in a course. The scores are provided to the student alone to plan for their growth in the program.

Assessment of learning [2,3]	An assessment designed to serve primarily as a tool to determine how well the student has learned the material or achieved a level of skill or competence.	Students in a program are given a certification exam to determine whether they have mastered the content in their specialty.

Assessment as learning [4]	Assessments are incorporated into the learning process itself; assessments serve to self-regulate learning, reinforcing knowledge and skill acquisition.	Students are provided with multiple-choice questions and artificial-intelligence-enhanced feedback highlighting strengths, areas for improvement, and opportunities for self-reflection [6].

Dual purposing [7]	Assessments are used for more than one purpose; typically, these purposes include a primary purpose and a secondary purpose, which are categorically different in their orientation toward promoting learning versus judging mastery of content.	Workplace-based assessments are used to guide learning during clinical rotations. These same data are then used at the conclusion of all rotations to determine whether students have met the competency threshold.

**Stakes**		

High-stakes [11]	The assessment is given significant weight within the education program, and thus, the consequences for high and/or low performance are significant.	Students are required to pass an examination to advance to the next stage of their training.

Low-stakes [11]	The assessment is given a relatively low weight within the program; thus, its consequences are relatively insignificant.	Workplace-based assessments are administered in the authentic clinical settings. The data from each assessment is used as one of many data points in evaluating a student’s performance.

**Purpose of assessment and learning**		

Formative [16,71]	An assessment designed to serve primarily as a tool to contribute to the development or formation of the learner. This is commonly designed to promote learning *during* an educational experience.	Students receive midpoint feedback on their performance during a clinical rotation.

Summative [16,71]	An assessment designed to serve primarily as a tool to determine how well the student has learned the material or achieved a level of skills or competence. This is commonly designed to measure learning acquired at *the conclusion* of an educational experience or where a progress decision is needed.	Students are evaluated on their performance at the conclusion of a rotation.

**Assessment systems**		

Program of assessment [72]	The use of multiple assessments within the larger educational program to create a cohesive system of assessment; alignment between assessments and purpose is sought.	A medical education program selects a variety of assessment methods; some are designed to assess knowledge, while others are targeted at assessing communication skills.

Programmatic assessment [2,26]	A specific program of assessment in which multiple data points are accumulated from multiple sources and then used to formulate a decision about a learner’s performance in a training program; focus is formative over summative; number of data points needed to make a decision is dependent on the stakes of the decision; a group makes high-stakes decisions of experts.	A competency-based program collects data from multiple-choice tests, workplace-based assessments, peer observations, and reflective writing assignments to determine whether a learner is competent for practice.


Both the purpose and the stakes of assessment lie on a continuum and are influenced, at least in part, by the learner’s perception [12,13,14]. For example, a clinical performance assessment (e.g., an in-training evaluation report) may be viewed primarily as a relatively high-stakes assessment of learning, since it is often administered after a rotation. However, that same evaluation may inform future learning if additional rotations remain, thus also serving as an assessment for learning and carrying less overall perceived weight in the bigger picture.

In competency-based medical education (CBME) and competency-based health professions education (CBHPE), herein referred to collectively as CBHPE, there is added complexity to both the purpose and the stakes of assessments. By definition, CBHPE programs adopt a programmatic assessment approach which requires aggregation of assessment data to formulate a comprehensive understanding of the learner’s competence [2]. In programmatic assessment, a given assessment may be designed to function primarily as a low-stakes assessment *for* learning while *also* serving as a component of a higher-stakes decision when used as one of many data points to inform a summative competency or entrustment decision. Therefore, the assessment serves more than one purpose and, similarly, portrays varying degrees of stakes depending on one’s vantage point [15]. This concept is described in the literature as “dual purposing” [7,8,9] a reflection of the competing and, at times, contradictory purposes for a given assessment. Given precedent in the literature, we also refer to this concept as *dual purposing*, while appreciating that assessments may have more than two purposes, and that purpose and stakes are not necessarily synonymous.

A classic example of dual-purposing involves collecting data from workplace-based assessments (WBAs). WBAs are defined as assessments obtained from observation of learners in the authentic work environment [16]. The primary purpose of WBAs typically involves guiding learners during their training (i.e., assessment *for* learning); however, a secondary purpose may be to use the same assessment data, as one component of an aggregated data set, to inform a summative determination regarding the learner’s progression toward competency attainment (i.e., assessment *of* learning). Figure 1 summarizes the relationship among purpose, stakes, and the impact of programmatic assessment approaches on how assessments are perceived.

**Figure 1:**
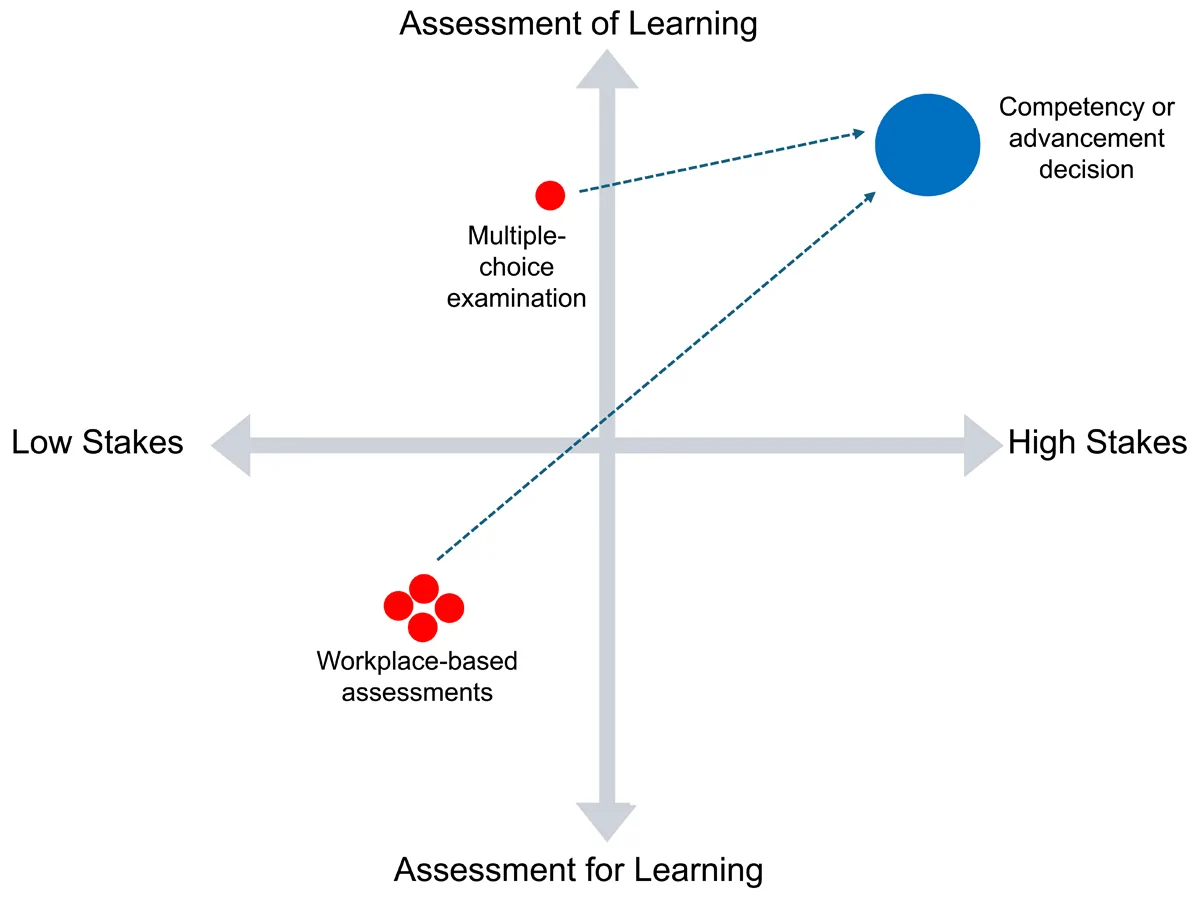
**The continuum of purpose and stakes of assessment with an illustration of “dual purposing” in a competency-based education program**. Legend: This figure illustrates two prototypical assessments commonly used in competency-based health professions education: end-of-course multiple choice examinations and workplace-based assessments. The red dot indicates the primary use of the assessment while the blue dot indicates a secondary use (i.e., “dual purpose”) of the assessment. This figure illustrates how a lower-stakes assessment can become a higher-stakes assessment (dotted arrow) when that assessment (and others) are used in aggregate to formulate a summative competency decision. Of note, this figure is meant to provide a hypothetical example; the shift in purposes is illustrative of the concept rather than exact.

### Dual purposing: the concerns and pragmatic considerations

Despite the potential alignment between dual-purposing and programmatic assessment approaches, authors have cautioned against this approach.9 Some studies suggest that dual purposing may undermine an assessment’s original intent, eroding the learner’s perception of “assessment *for* learning” [17,18,19]. Moreover, dual purposing raises theoretical and practical challenges for educational leaders. A key consideration when examining the validity of any assessment is the intended use of the assessment data (i.e., the stakes) [20]. How does one reconcile an assessment that is both high and low-stakes?

Despite these concerns, there are times when dual purposing may be necessary. Issues such as supervisor buy-in, time constraints, and competing patient care responsibilities are commonly cited barriers limiting the collection of meaningful and sufficient WBA data [7]. For these reasons, some may consider the development of two discrete systems of assessment, one purely *for* learning while another purely *for* learning, impractical and unrealistic. Some further argue that differentiating between purposes is more an academic exercise than a meaningful distinction. The literature suggests, for example, that learners perceive all assessments as measures *of* learning, even when their stated purpose is different [12,13].

In the remainder of this manuscript, we explore dual purposing of assessment in CBHPE programs from a pragmatic viewpoint, offering three primary design conditions to maximize benefits while minimizing the risks: 1. Setting the stage by creating a trustworthy culture, 2. Obtaining and interpreting the data by leveraging programmatic assessment and technology, and 3. Using the data to promote growth and competency attainment by harnessing learner agency. These perspectives are explored below and summarized in Table 2.

**Table 2:** Key considerations for effective “dual purposing” of assessments in a competency-based health professions education program.


KEY CONSIDERATION	HOW TO DEVELOP THE COMPONENT	RISKS IF COMPONENT IS ABSENT

**Setting the stage**		

Culture	Foster and normalize growth-orientation and likelihood of failures with explicit encouragement and possibly even requirements to include documentation of both in progress and successful workplace-based observations Develop faculty and learners’ orientation to a culture of growth through dedicated and possibly co-delivered sessions Build safe, trusting, collaborative relationships among learners, supervisors and program leaders Ensure processes for how, when, and why assessment data will be used are made transparent to all	Continued performance-orientation where learners may perceive all assessments as high-stakes hurdles rather than opportunities for growth, self-reflection, and improvement Avoidance of observation and feedback of clinical encounters during expected developmental phase of learning Increase learner stress regarding fear of being identified/labelled as low performing Reinforces supervisors’ reluctance to document honest corrective feedback

Communication	Outline roles and responsibilities for all constituent groups Share policies, procedures, standards, and expectations for assessment data with learners, supervisors, longitudinal advisors, and educational leaders Ensure alignment of growth-oriented practices across learner, supervisor, program, and competence committee/summative decision makers to avoid undermining of trust	Result in supervisor feedback that is not aligned with intended goals Result in lack of completion of expected coaching feedback and assessment activities by involved members with lost opportunities for learner development and support Creates hidden curriculum messaging that seeking developmental assessment for learning is not valued or safe in the program Lack of a shared mental model

**Obtaining/Interpreting the data**		

Programmatic assessment	Gather multi-modal data from multiple, diverse sources	Result in incomplete, non-representative, or biased data informing competence decisions

Lower-stakes assessment	Minimize purely summative high-stakes assessments for decision-making Incorporate opportunities for providing feedback even in high-stakes settings	Lead to a less comprehensive, authentic picture of learner performance Risk high-stakes assessments being seen as “hoops to jump through” without meaningful learning Promote low volume data collection at risk for selection of cases that are not representative of usual performance (selection bias)

Opportunities for data collection	Provide sufficient opportunities for supervisors to observe learners and to collect assessment data and provide actionable suggestions for improvement When observation is not possible, reconsider the feasibility and/or value of obtaining the data or provide alternate means for learners to demonstrate competence in these areas Shared responsibility of the clinical supervisors and learners to trigger an assessment	Risk low volume data collection, which can increase the relative stakes and pressure to perform for available observations/cases Increase learner stress as they struggle to find opportunities to demonstrate competence for rarely occurring yet required clinical contexts

Technology	Leverage new and emerging technology to facilitate the collection, aggregation, and interpretation of assessment data Critically appraise technology for its potential to promote excessive surveillance and meaningful data interpretation Consider ethical considerations and consent requirements in accessing patient care data	Decrease trust and increase learner stress due to continuous assessment Increase administrative burden of programs and competence committees to prioritize, interpret, and integrate large volumes of collected data into assessment of competence Create concern regarding patient privacy and information safety

**Learner agency**		

Learner-initiated assessment	Promote balanced opportunities for learners and supervisors to initiate assessment	Promote passive learner role with diminished learner motivation and autonomy or place excessive burden on residents to collect data without consequences for supervisors who don’t reciprocate Decrease learners’ ability to seek feedback at times when they need direction to practice lifelong learning skills Lack accountability of learners and supervisors if the expectation to initiate assessments is not shared

Coaching relationships	Encourage coaching approaches in any feedback conversations (e.g. following observation in clinical settings or case presentations, mid-point and end-of-rotation discussions, periodic review of learning portfolio) Develop longitudinal coaching programs to support learners in their growth and development and their lifelong learning skills throughout the program	Create perceptions of assessment moments as meaningless, low-yield “checkbox” activities Miss opportunity for guided self-reflection and development of lifelong learning skills Result in learners unsure where to focus their efforts to maximize their development and frustration with lack of progression

Ultra-low-stakes assessments	Consider learner-identified, developmental-only assessment data that may be used for growth Carefully consider who will see this data and ensure explicit, transparent guidelines for when it may be disclosed to summative decision makers	In low trust settings, learners and/or supervisors may hide significant concerns or patient safety issues through this system Learners or supervisors may feel betrayal of trust if critical safety data is disclosed to summative decision makers without transparent communication of the expectations for its release If patterns of significant deficits are not disclosed, programs may not have sufficient data to offer learner supports and to protect the public


## Setting the stage: Creating a trustworthy culture

### The importance of culture

CBHPE is inherently focused on growth throughout training. A growth-orientation implicitly anticipates errors, continuous improvement, and longitudinal development toward competency attainment [21,22]. The notion that learners may, and often will, struggle represents a point of tension in CBHPE, where the reality of “fallible learning” [23] is at odds with a prevailing expectation of “infallible competence” [23] in HPE culture [23,24] In the absence of trust, learners may perceive all assessments as high-stakes hurdles rather than opportunities for growth, self-reflection, and improvement [25,26], thus hindering any efforts to promote acquisition of competency. Navigating this tension requires an educational alliance between the learner, their supervisors, and the educational leadership team, empowering learners to take advantage of opportunities for feedback and growth [27]. Creating explicit opportunities to build safe, collaborative relationships between learners, supervisors, and program leaders is crucial; these alliances must normalize and support a growth-oriented approach to learning.

### Fostering a culture of trust

Efforts at the individual, interpersonal, and program/institutional level are likely required to support a culture of trust. Learners should receive training on a growth mindset and incorporating feedback into practice. Faculty development should target feedback dialogue, ensuring that assessors are trained to provide specific, actionable comments and encouraging supervisors to cultivate safe, meaningful relationships with learners through role modeling, benevolence, and collaboration on shared goals [27,28,29,30,31]. Fostering a learning environment with open dialogue—where learners are empowered to seek clarification, co-construct learning goals based on feedback, and develop action plans—reinforces trust in the assessment system and promotes a growth mindset [9,32].

At the program level, a culture of trust requires well-articulated and transparent processes and procedures that dictate how, when, and why assessment data will be used throughout a learner’s education, including the roles and responsibilities of all constituents (e.g., learners, supervisors, educational leaders, competency committee members, etc.), and how decisions will be shared throughout the developmental process. Embedding regular low-stakes assessments for learning encourages reflection and iterative improvement [26,33] Portfolios that incorporate programmatic assessment approaches combined with longitudinal mentoring and/or coaching structures are another tangible method for ensuring an appropriate infrastructure to support competency attainment [2]. Such systems ensure that learners are provided continuous feedback for learning and also aggregation of longitudinal data to support summative decision-making. Onboarding and regular reinforcement are necessary strategies to ensure shared mental models among faculty responsible for all aspects of a learner’s assessment.

### Communicating the dual purposes to key constituents

Finally, it is critical to consider how to communicate policies, procedures, standards, and expectations for assessment. As previously described, even when assessments are described as “low-stakes,” “formative,” or “for learning,” learners may not perceive them as such.^12^ As a consequence, we suggest that rather than label assessments for one purpose or the other, it may be more appropriate to outline exactly *how, when, and why* assessments will be used for each respective purpose. Such communication may galvanize learners to become active members in the learning process^28^ and may yield comments and/or ratings from supervisors that are better aligned with programmatic assessment goals. These standards should be shared publicly through both written (e.g., policies, procedures, etc.) and verbal (e.g., meetings within departments, schools, etc.) communications. Collectively, this may result in better growth opportunities for learners, improved satisfaction among raters, and more confidence in summative competency decision-making at the program level.

## Obtaining and interpreting the data: Leveraging programmatic assessment and technology

### Adopting a programmatic assessment approach

Programmatic assessment, a core component of CBHPE [34], also serves as an invaluable method for ensuring the effective dual-purposing of assessment data. Programmatic assessment is a comprehensive concept comprising several components, yet its central focus is the collection of large amounts of data from diverse sources [26]. Cees van der Vleuten, the architect of programmatic assessment, frequently framed it through the analogy of pixels in a picture [35]. For example, in a large impressionist painting, if we zoom in, we may see unexpected or idiosyncratic color differences. However, if we zoom out, the entire picture becomes clear, and the small deviations at the individual-pixel level become less meaningful.

The same is true when considering a learner’s progression toward competency attainment in a programmatic assessment model. Individual assessments offer an imperfect snapshot of competence, which may introduce noise into the data. For these reasons, each data point carries little value on its own in making high-stakes decisions. However, when assembled into a larger picture, the collective data provides a clearer picture of performance, again aligning with the key features of programmatic assessment [26,36]. As a consequence, we may accurately suggest that *individual* data points can be viewed as primarily for learning (i.e., one purpose), while the entire body of assessment data can be used to determine whether competency has been achieved (i.e., a second purpose).

Programmatic assessment requires not only a large quantity of data but also data from multiple sources and types. If we consider the aforementioned painting analogy, we may view the use of multiple assessment strategies akin to the value color adds. Compared to a grayscale painting, one with color can provide a more realistic impression of the world around us. Adding assessment data from diverse sources provides a more comprehensive and realistic picture of the trainee’s performance. Though programmatic assessment is complex, guides to support educational leaders are available, including a recently published, practical four-step process [37].

### Increasing the use of lower-stakes assessments

Traditional approaches to assessment in health professions education have largely emphasized the summative function of assessment of learning through episodic, high-stakes examinations [38]. An example involves using a single multiple-choice test to determine whether learners are deemed competent for practice. When used exclusively for summative purposes, such high-stakes assessment fails to capture the complexity and longitudinal nature of competency development and limits opportunities for feedback [39]. However, extensive research by Harrison and colleagues demonstrates that, when integrated within a culture of learning and feedback, high-stakes assessments, such as objective structured clinical examinations (OSCEs), can serve as both instruments *of* and *for* learning [19,40,41,42].

It is therefore critical to incorporate both high *and* low(er)-stakes assessments into a programmatic assessment approach. Lower-stakes assessments may include those obtained within the workplace (i.e., workplace-based assessments, WBAs). Regular, low-stakes WBAs embedded within authentic clinical work provide opportunities for feedback while also offering rich data to inform a summative competency decision, thus serving as a classic example of an assessment for multiple purposes [43]. Though the volume of assessments obtained from WBAs may raise concerns about assessment fatigue, a regular cadence of assessments embedded in clinical work, coupled with engaged supervisors, can foster a learning-oriented environment where observation and feedback are normal and expected parts of training. As the number and diversity of data points increase, the draw to use any single assessment encounter to inform high-stakes decisions, which is anathema to programmatic assessment, decreases [9].

### Providing opportunities to collect data

Though programmatic assessment provides a system to reduce the emphasis on any one data point, the success of this approach is inherently dependent on sufficient clinical opportunities. For example, suppose a learner is required to demonstrate competence in prescribing medications for a breastfeeding person. However, that same learner rarely has an opportunity to see breastfeeding patients. In this context, any observations may be perceived as inherently high-stakes, since they represent rare opportunities to collect assessment data for the specific competency.

To mitigate this risk and uphold the principles of programmatic assessment, programs should carefully consider the feasibility of the required assessments. In some cases, there may be an appropriate rationale for requiring demonstration of competence of a rarely encountered clinical skill. In these cases, we still recommend using the principles of programmatic assessment to ensure a variety of opportunities and assessment strategies are available to demonstrate competence. However, the program may need to rely more heavily on standardized assessment environments to obtain assessment data. For example, the program may arrange a simulation session with supervisors available to complete observations and include the topic in a structured oral exam or an OSCE. Within the context of skills such as these, the role of dual-purposing remains paramount; supervisors may provide formative feedback in the standardized setting and then use the same data to inform a summative decision at a later time [19,40].

Further, incorporating learning opportunities into an orientation session could help learners acquire a baseline comfort/competence. This may then allow any future encounters with the rare situation to refresh a learner’s baseline competency and provide an opportunity for further development toward mastery. Certification courses (e.g., advanced life support training) can also help serve this role by decreasing reliance on WBA for rare clinical encounters. Finally, programs may advocate scheduling learners in clinical settings that specialize in the contexts in need, which may not be as easy for them to access routinely.

### Leveraging the value of technology

The collection and interpretation of large volumes of data to support a CBHPE program are often challenging. For this reason, existing and emerging technologies can be leveraged. For example, mobile technologies allow for rapid collection and synthesis of WBA data to provide feedback to learners and facilitate summative competency decisions [44] Many programs are now using such technology, and examples are pervasive in the HPE literature [45,46,47,48]. More recently, a myriad of low-touch (i.e., little human effort needed for collection) technology-based solutions [49] have been developed in the clinical arena. These advances integrate data from clinical care directly into assessment portfolios, thus bypassing the need for human interaction.

An important emerging example of low-touch technology involves ambient AI, a method of data collection that relies on audio/video recording or motion capture (e.g., haptics) to capture clinical performance data without the need for human observers. Current examples, which may be collected with or without AI support, include procedural haptics, learner time-motion tracking, eye-tracking during patient encounters or while reading diagnostic studies, and recording verbal communication during code simulations [49,50,51,52].

Each of these technologies holds promise to generate quantities and types of data that were previously unattainable [53], thus mitigating concerns about the efficiency of dual-purposing.

Critically though, these solutions have downsides. One major risk involves the perception of a surveillance state; if every utterance, procedural movement, keystroke, or electronic health record measure is translated into an assessment, learners may feel as though each action is a performance-based judgment. The volume of data could overwhelm a competency committee and learners, thus reducing the value of assessment and necessitating training and increased use of coaches to help learners interpret data meaningfully. Patient consent issues for educational purposes of this data also need to be considered. For these reasons, it is important to think critically about technology; it offers significant promise to address challenges with data collection but may also introduce other challenges as a side effect. Educators should demonstrate humility around innovative technologies and recognize the importance of governing their use through a trust-based system [54].

## Using the data to promote growth and competency attainment: harnessing learner agency

To this point, we have considered the structural supports required to optimize a dual-purposing assessment program and the mechanisms for data collection and aggregation that promote this process. In a CBHPE program, instruction is individualized to the specific needs of the learner [34]. In this section, we turn to the final design condition recommended to optimize dual purposing of assessments; namely, how to translate data into action to support learner growth and identify individual needs. We specifically focus on the learner’s role and agency in using data for their own growth and development within a system that uses dual-purposing of assessment.

### Learner-initiated assessment

In traditional assessment programs, assessments are driven by the educational program leadership. Relying solely on such approaches limits learner agency and, thus, may diminish motivation and autonomy during the learning process [55]. Facilitating learner-initiated assessment is therefore one key mechanism to support learner agency and assessment for learning in a system of dual-purposing assessment. The literature demonstrates that, when learners lead feedback, goal setting, and action planning, their engagement increases and behavior change is more likely [56,57,58,59]. However, learners may still hesitate to initiate assessment due to fears of burdening supervisors or disrupting workflow [28,60] This hesitation is intertwined with the vulnerability and emotional burden of seeking observation and feedback, which requires openness and reflection [23]. Therefore, we again return to highlight the critical role that culture and trust play in the risk-benefit equation in dual-purposing of assessment. Strengthening emotional bandwidth through supportive, trusting environments can enhance learners’ capacity to engage meaningfully with feedback [23].

Clinical supervisors can foster learner agency by explicitly and regularly reminding learners that daily interactions are primarily formative rather than summative. Consistently prioritizing *learning moments* over higher-stakes *assessment moments* reduces anxiety and nurtures a growth-oriented culture, thereby empowering learners to take ownership of their learning [61,62]. Learners can advocate for their own success in myriad ways, including self-education about the goals, nomenclature, and design of their CBHPE program, as well as understanding the value of a growth mindset [63]. Importantly, we would advocate that the learner should initiate at least some assessments. However, it is important to share this initiation activity with supervisors to reduce the burden on learners and ensure that observations are representative of a learner’s progress. Encouraging learners to articulate and pursue their own goals fosters self-direction and ownership of development [57], Framing learners as active participants in their learning (and assessment) reinforces this agency, thus mitigating some concerns about dual-purposing [27].

### Coaching relationships to support longitudinal growth

Strong educational relationships are essential for promoting learner agency and overcoming the many intrinsic barriers associated with feedback and assessment cultures. Effective coaching relationships are built on trust, mutual respect, and shared responsibility [31,64]. Longitudinal clinical teaching structures (e.g., longitudinal clinics, longitudinal supervisor relationships, etc.) can provide these opportunities and promote honest dialogue and more frequent feedback-seeking [60]. Within such relationships, feedback becomes a collaborative, psychologically safe process aligned with the educational alliance model [65] transforming WBAs from assessments into concurrent opportunities for joint growth and continuous development [57]. Importantly, coaching approaches may extend to relationships that are more time-limited as well. For example, the relationship, reaction, content, and coaching (i.e., R2C2) model is a well-described and successful mechanism for incorporating coaching systems into mid- and end-of-clinical-rotation feedback sessions [66,67,68]. Regardless of the model, embedding structured coaching sessions in which learners review their own aggregated data is an important element in supporting longitudinal growth.

### Considering “ultra-low-stakes” assessments and learner agency

One final concept worth discussing concerns developmental-only, or so-called “zero-stakes,” assessments [9]. The concept behind this idea is that some data may be used purely for learning purposes, thereby promoting learner agency in the assessment process. This concept aligns with broader recommendations for integrated assessment designs to uphold both learner development and agency as well as institutional accountability [14,69,70].

However, several tensions and concerns arise with zero-stakes proposals. For one, any notion of zero-stakes assessment must be carefully considered to maintain accountability to the public. Who is permitted to see this data (e.g., learners only, key supervisors, longitudinal coaches, mentors, etc.) and for what purposes? What might/should supervisors or longitudinal coaches do if they note recurrent themes of learner or patient safety concerns identified in zero-stakes assessment? These tensions between learner development and accountability to the public thus exemplify the practical concern with zero-stakes assessment. For these reasons, guardrails need to be considered to delineate which information from such assessments *must* be fed forward for further consideration and to inform learner support or summative decisions potentially. For example, are there patient safety concern thresholds for which the decisions to share data with program leaders and/or include it in the learner portfolio should overrule the learner’s discretion or preference? Do learners and supervisors clearly understand the criteria that would allow the disclosure of these assessments for further consideration?

A bigger question is whether there is such a thing as true zero-stakes assessments. When a learner is assessed, there is always some element of stakes (real or perceived). Perhaps it is therefore better to consider assessments on a spectrum, with the lowest end best described as “ultra-low-stakes” assessments, rather than truly zero-stakes assessments. Further, by creating shadow systems, programs are indirectly signaling that ultra-low-stakes feedback cannot be safely and thoughtfully considered by competency committees or program leadership. This risks reinforcing the unintended message that learners will be penalized for including valuable evidence of progression through early developmental observations in their portfolios, thereby privileging performance over mastery-based, CBHPE-focused approaches to learning.

Finally, in systems where supervisors and learners are already highly overextended by clinical demands, it may not be realistic to implement additional robust systems that can ensure both the collection of sufficient documented programmatic assessment data and shadow feedback documentation to inform the dual purposes of progress decision-making and developmental support. Overall, even if true “zero-stakes” assessments are not possible, opportunities to limit the stakes may help support learner agency. Educators should identify opportunities when possible and incorporate these into their assessment programs.

## Maximizing Success, Minimizing Risks, and Caveats to a Dual Purposing Strategy

We recommend design approaches to maximize the success of dual purposing but recognize that failure to implement practices consistent with these approaches at every level has associated risks. For example, extensive faculty/learner development is valuable to foster a culture of growth and trust. However, if the competency committee penalizes learners who embrace a mastery approach, a hidden curriculum will emerge, suggesting that engaging in assessment-for-learning moments is neither valued nor safe in the program. Similarly, a program should develop systems that support low-stakes workplace-based observations. Yet, if program structures and supervisor accountability are not in place to allow learner observations to be collected at a regular cadence and in adequate volume, then the few successfully collected observations will serve as higher-stakes observations that contribute in a substantially higher weighting than intended to summative decision making. A summary of these approaches and risks is provided in Table 2.

Finally, although this manuscript is centered on the premise that dual-purposing of assessments is often a practical necessity, many factors contribute to the decision to proceed with dual-purposing. Leaders should be critically reflective in appraising their institutional environment. What is the institutional commitment to promoting a culture of trust, developing technology support programs, or longitudinal relationships? The misalignment between the institutional mission and the infrastructure that supports dual purposing is problematic. And the counter is also true: “Is dual purposing necessary?” Some programs may have resources (e.g., personnel, finances, technology) to support separate assessment systems, thus negating the need for dual-purposing.

## Conclusions

In this manuscript, we explored the prospect of using assessments for more than one purpose (i.e. ‘dual purposing’) within a CBHPE program. Though such an approach is potentially problematic, it is also a practical reality in many health professions education programs worldwide. As we outlined, dual purposing requires diligence to ensure a well-designed cultural foundation, systems for sufficient data collection, and involvement with the learner to provide a simultaneous balance between assessment for and of learning, maximizing learner growth and upholding accountability to our health care professions. By strategically incorporating several design principles (e.g., building explicit structures of trust, generating high-volume aggregate data, and supporting learner agency), we can ensure that dual-purposing can serve as a strategic integration of formative and summative functions, rather than a compromise of both.
